# Conservation of immune gene signatures in solid tumors and prognostic implications

**DOI:** 10.1186/s12885-016-2948-z

**Published:** 2016-11-22

**Authors:** Julia Chifman, Ashok Pullikuth, Jeff W. Chou, Davide Bedognetti, Lance D. Miller

**Affiliations:** 1 Department of Cancer Biology, Wake Forest School of Medicine, Medical Center Boulevard, Winston-Salem, 27157 NC USA; 2 Department of Biostatistical Sciences, Wake Forest Public Health Sciences, Medical Center Boulevard, Winston-Salem, 27157 NC USA; 3 Sidra Medical and Research Center, Doha, Qatar

**Keywords:** Consensus clustering, Enrichment scores, Immune signatures, Survival analysis

## Abstract

**Background:**

Tumor-infiltrating leukocytes can either limit cancer growth or facilitate its spread. Diagnostic strategies that comprehensively assess the functional complexity of tumor immune infiltrates could have wide-reaching clinical value. In previous work we identified distinct immune gene signatures in breast tumors that reflect the relative abundance of infiltrating immune cells and exhibited significant associations with patient outcomes. Here we hypothesized that immune gene signatures agnostic to tumor type can be identified by de novo discovery of gene clusters enriched for immunological functions and possessing internal correlation structure conserved across solid tumors from different anatomic sites.

**Methods:**

We assembled microarray expression datasets encompassing 5,295 tumors of the breast, colon, lung, ovarian and prostate. Unsupervised clustering methods were used to determine number and composition of gene clusters within each dataset. Immune-enriched gene clusters (signatures) identified by gene ontology enrichment were analyzed for internal correlation structure and conservation across tumors then compared against expression profiles of: 1) flow-sorted leukocytes from peripheral blood and 2) >300 cancer cell lines from solid and hematologic cancers. Cox regression analysis was used to identify signatures with significant associations with clinical outcome.

**Results:**

We identified nine distinct immune-enriched gene signatures conserved across all five tumor types. The signatures differentiated specific leukocyte lineages with moderate discernment overall, and naturally organized into six discrete groups indicative of admixed lineages. Moreover, seven of the signatures exhibit minimal and uncorrelated expression in cancer cell lines, suggesting that these signatures derive predominantly from infiltrating immune cells. All nine immune signatures achieved statistically significant associations with patient prognosis (*p*<0.05) in one or more tumor types with greatest significance observed in breast and skin cancers. Several signatures indicative of myeloid lineages exhibited poor outcome associations that were most apparent in brain and colon cancers.

**Conclusions:**

These findings suggest that tumor infiltrating immune cells can be differentiated by immune-specific gene expression patterns that quantify the relative abundance of multiple immune infiltrates across a range of solid tumor types. That these markers of immune involvement are significantly associated with patient prognosis in diverse cancers suggests their clinical utility as pan-cancer markers of tumor behavior and immune responsiveness.

**Electronic supplementary material:**

The online version of this article (doi:10.1186/s12885-016-2948-z) contains supplementary material, which is available to authorized users.

## Background

Immune cells that traffic to solid tumors can exert profound influences on the clinical behavior of cancer. Tumor-infiltrating immune cells such as cytotoxic T lymphocytes (CTL), T-helper (T _H_) cells, natural killer (NK) cells and dendritic cells (DC) are generally known to effect anti-tumor immune responses that can limit tumor growth and progression, while others such as T-regulatory cells (T-reg), tumor associated macrophages (TAM) and myeloid derived suppressor cells (MDSC) are associated with pro-tumorigenic functions that disable anti-tumor immunity and facilitate cancer invasion and metastasis. Consistent with their functional attributes, these various immune cell types have been shown to confer clinically-relevant prognostic information predictive of either good or poor patient outcomes depending on cell type, abundance and functional orientation. However, for reasons that remain unclear, immune prognostic value is known to vary according to tumor site and histology, and is likely impacted by signals intrinsic to the tumor microenvironment including factors expressed by cancer cells or other immune cells with antagonizing functions. New diagnostic strategies that comprehensively and simultaneously assess the cellular composition and functional complexity of immune infiltrates in solid tumors is needed. Such a diagnostic systems level view of tumor immunity could markedly enhance patient prognosis and inform immunotherapeutic decisions for cancer patients. Conventional strategies for assessing immune involvement in cancer are limited in this capacity. For example, tumor infiltrating lymphocytes (TIL) are readily observable in tumor sections by conventional histological staining methods, and their relative abundance has, historically, been widely associated with good clinical outcomes in multiple cancer types including breast, colon, lung, ovarian and skin cancers [1–5]. TIL assessment, however, lacks objective quantitation and is subject to the inherent limitation of cellular heterogeneity, namely a lack of discernment among the varying types and proportions of immune cells that together comprise TIL [6], prompting the formation of international consortia to develop standardized methods for TIL evaluation [7]. By contrast, immunohistochemical (IHC) methods that stain for immune cell-specific markers offer greater accuracy and precision for quantifying biologically distinct immune populations, but practical limitations associated with IHC such as reagent costs and labor, prevent the comprehensive (multi-cellular) assessment of the immune contexture of tumors on a routine basis, though new multispectral imaging approaches are beginning to show promise [8].

While a number of different immune signatures have been reported, there remain obstacles to their clinical translation. For example, the genetic composition of reported immune signatures has been mostly inconsistent, varying widely within and across tumor types. The ability of these genes to discern specific immune cell lineages is poorly understood. How malignant cells contribute to the expression of these genes in a manner that may obscure their immune-specific origins has not been systematically addressed.

Herein, we investigated the hypothesis that immune cell signatures agnostic to tumor type could be identified by the de novo discovery of gene signatures comprised of genes enriched for immune biological functions and with internal correlation structure conserved across solid tumors from different anatomic sites. We identified nine distinct immune gene signatures with fully conserved correlation structures in breast, lung, colon, ovarian and prostate tumors that differentiated specific leukocyte populations to variable degrees. These signatures also exhibited significant statistical associations with patient prognosis while presenting some substantial differences among various cancer types. Together, these findings indicate the existence of tumor-agnostic immune-specific gene signatures that appear to quantify a variety of immune cell lineages with prognostic implications for cancer patients.

## Methods

### Cancer microarray datasets used for identification of immune gene signatures

To discover immune-related gene signatures in human tumors, we assembled five curated microarray datasets of primary tumor expression profiles for breast, colon, lung, ovarian and prostate cancers. All five datasets are based on the Affymetrix U133 GeneChip microarray platform with specific array platforms: HG-U133A, HG-U133A2 and HG-U133 PLUS 2.0. Only probe sets in common to all gene chips were included for analysis, which resulted in 22,277 probe sets.

Each cancer dataset represents a compilation of multiple smaller tumor profiling datasets. The breast cancer dataset is described in detail in [9]. It consists of 2,034 primary invasive breast tumors from multiple medical centers in the U.S., Europe and Asia. The colon cancer dataset consists of 843 tumor profiles derived from four studies. Raw data was downloaded from NCBI Gene Expression Omnibus (GEO) database [10, 11] (accessions: GSE26682, GSE17538, GSE14333, and GSE13294). The non-small cell lung cancer dataset consists of 1,346 samples from 11 studies. Eight of them were extracted from GEO (accessions: GSE10072, GSE10245, GSE10445, GSE19188, GSE31210, GSE3141, GSE31908, and GSE4573). One dataset was downloaded from NCI caArray microarray data repository http://cabig.cancer.gov/solutions/applications/caarray/ (accession number: jacob-00182) and is now available on GEO: GSE68465. Additionally, this dataset contains unpublished samples: 77 samples (Paris series II; Dr. Philippe Broet, by communication) and 50 samples (Singapore; Dr. Patrick Tan, by communication). The ovarian cancer dataset consists of 740 tumor profiles from six studies. Raw data was downloaded from GEO database (accessions: GSE18520, GSE26193, GSE26712, GSE27943, GSE6008, and GSE9899). The prostate cancer dataset consists of 332 tumor profiles from three studies. Raw data was downloaded from GEO database (accessions: GSE17951, GSE25136, and GSE8218).

Each dataset (breast, colon, lung, ovarian and prostate) was processed on individual study using the Robust Multi-array Average (RMA) method that includes background correction, quantile normalization and summarization. RMA processing is implemented in the R [12] package *affy* [13] as provided by Bioconductor [14]. Batch effects were corrected using ComBat, an Empirical Bayes method [15].

### Data filtering using EPIG

To extract major patterns of genes in our five datasets (described above) we have used EPIG, which is a method for **E**xtracting Microarray Gene Expression **P**atterns and **I**dentifying co-expressed **G**enes [16]. Prior to EPIG analysis, we averaged expression (log2 signal intensities) of probe sets that corresponded to the same gene with a Pearson *r*-value greater than 0.4. Next, for each dataset 50% of samples were randomly selected and the EPIG algorithm was applied to extract major patterns of co-expressed genes. This process was repeated 1000 times. For each cluster we chose genes that were selected 750 times or more out of 1000. Gene-annotation enrichment analysis using the Database for Annotation, Visualization and Integrated Discovery (DAVID) [17, 18] was performed on all final clusters. Clusters of genes that were highly enriched (*p*<0.001) for immunity-related terms were selected for further analysis. At this stage we went back to individual probe identifications and took the union of all probes among five datasets resulting in 1,017 Affymetrix probe IDs.

### Consensus clustering

We have selected two different unsupervised clustering methods for analysis of datasets (described above) each containing 1,017 probe sets: self-organizing maps (SOMs) [19–21] and *k*-means [22–25]. To assess cluster stability we further adopted the consensus clustering methodology of Monti et al. [26]. In addition, two different environments that employ consensus clustering technique were used: *ConsensusClustering* module implemented by Monti et al. [26] in GenePattern [27], and the package *clusterCons* implemented by Simpson et al. [28] in R [12]. We have used SOMs with the GenePattern module *ConsensusClustering* and *k*-means with R package *clusterCons*.

The consensus clustering procedure begins by specifying the range of clusters to be investigated and the clustering algorithm, i.e., k-means, or self-organizing map (SOM). Next, a proportion of genes or samples from a dataset is selected and clustered by using the specified algorithm and other parameters. This process is repeated many times and clusters produced by each iteration are stored and then used to calculate the consensus results. Genes that are recurrently identified in the same cluster can be deemed reliable cluster members. We have chosen the maximum number of clusters to investigate to be 10, and run 500 resampling iteration for both algorithms with 80% of probe sets being subsampled from the 1,017 probes without replacement.

Several objects and summary statistics are computed that can be used to assess the clusters’ composition and to quantify the stability of each cluster. One of the main objects is the *consensus matrix* that measures the frequency with which any two probe sets cluster together. We can rearrange items in the consensus matrix that belong to the same cluster and display it as a heatmap. In the event of a perfect consensus the heatmap will have sharply colored blocks along the diagonal. Other summary statistics are *cluster* and *item* consensus, which can be used to quantify the stability of each cluster, and to rank items within clusters in terms of how representative of a given cluster they are.

### Enrichment scores

Enrichment scores were computed using the immune cell profiling dataset of Abbas et al. [29] downloaded from the NCBI Gene Expression Omnibus database [10, 11], accession GSE22886. Expression data (Affymetrix HG-U133A) was processed using RMA as implemented in the R [12] package *affy* [13] and provided by Bioconductor [14]. We partitioned this dataset into 18 groups representing specific immune cell subsets (see Table 1). To compute enrichment scores for each probe set per group we have used the procedure as described in [30] and *limma* package of Bioconductor [14, 31, 32]. The procedure can be summarized as follows: first, one compares each group to all others and computes the linear model coefficient for each pair, which is a measure of the difference between two groups, then for each probe set one sums all linear model coefficients with *p*≤0.05 (Bonferroni corrected).

**Table 1:** Immune cell subsets

Immune descriptor	Replicates
CD8Tcell-N0-1	4
CD4Tcell-N0-1	3
CD4Tcell-Th1	5
CD4Tcell-Th2	6
MemoryTcell-RO-unactivated	3
MemoryTcell-RO-activated	3
NKcell-control	4
NKcell-IL2-stimulated	5
NKcell-IL15-stimulated	6
Bcell-naïve	7
Bcell-Memory	8
PlasmaCell	7
Monocyte-Day0	12
Monocyte-Day1	12
Monocyte-Day7	12
DendriticCell-Control	6
DendriticCell-LPS-stimulated	6
Neutrophil-Resting	5

### Gene-annotation enrichment analysis

Gene-annotation enrichment analysis using the Database for Annotation, Visualization and Integrated Discovery (DAVID) [17, 18] was performed on all final meta-intersections (see Results section for definition of meta-intersection). In selecting the candidates that will become signatures we have used the following criteria: (i) at least 50*%* of probe sets in each meta-intersection had to be annotated for GO biological process and function, (ii) there must be at least ten unique gene symbols and titles in each intersection, and (iii) from the remaining meta-intersections we selected only those with significant enrichment (FDR <0.05) for immune functions.

### Metagene construction

The construction of immune metagenes was performed as follows. First, for each cancer dataset (described above) we averaged probe sets within a metagene that represent the same gene to ensure that no gene is overrepresented. Next, the signal intensities of the genes from the first step and intensities of the remaining probe sets were averaged to form a final metagene.

### GSK cell lines data

Expression data (Affymetrix HG-U133 PLUS 2.0) from over 300 cancer cell lines provided by GlaxoSmithKline (GSK) was processed using RMA as described in the previous sections. This dataset contained three technical replicates per cell line. After processing we averaged the replicate data (per cell line) which resulted in 318 samples. The dataset can be downloaded from National Cancer Institute’s caArray Directory https://wiki.nci.nih.gov/display/caArray2/caArray+Directory (Experiment ID: woost-00041).

### Datasets for survival analysis

For survival anlysis we have used six datasets that were annotated with survival time and event. Three of these datasets are subsets of the data described above and three are from The Cancer Genome Atlas (TCGA) Research Network: (http://cancergenome.nih.gov/).

#### Data used for metagene discovery

The breast cancer dataset contains 1,954 cases (out of 2,034) annotated with distant metastasis-free survival (DMFS) time (years) and event. For more information about breast cancer dataset clinical annotations consult [9]. For the colon dataset we have used GEO accession GSE17538 [33, 34]. This data contained patient and clinical characteristics. Of these, 232 cases were annotated for overall survival (OAS) time and event, 177 cases were annotated for disease specific survival (DSS) time and event, and 200 cases for disease free survival (DFS) time and event (all times are in months). Lung cancer dataset consists of 757 cases (out of 1346) annotated for overall survival (OAS) and progression-free survival (PFS) time and event, and 507 cases for relapse-free survival (RFS) time and event (times are in years).

#### TCGA data

Glioblastoma multiforme (GBM) and Ovarian serous cystadenocarcinoma (OV) Level 1 raw data (Affymetrix HG-U133A) and clinical information were downloaded from the TCGA data portal (https://tcga-data.nci.nih.gov/tcga/). Raw data was grouped by Plate ID and processed using RMA as implemented in the R [12] package *affy* [13] and provided by Bioconductor [14]. Batch effects were corrected using ComBat [15], which is part of the package *sva* [35]. Arrays that did correspond to the same patient were removed prior to preprocessing. The OV dataset had 566 cases and the GBM dataset had 524 cases annotated for overall survival (OAS) time (days) and event.

Skin Cutaneous Melanoma (SKCM) Level 3 data (RNASeqV2 normalized results for expression of a gene) was downloaded using R based data client (RTCGAToolbox [36]) for Firehose [37] pre-processed data. The SKCM dataset had 456 cases annotated for overall survival (OAS) time (days) and event.

### Survival analysis

Cox proportional hazards model (*survival* package [38, 39] as implemented in R [12]) was fitted to each dataset described above (Datasets for statistical analyses) using each metagene individually as continuous explanatory variable. To deal with tied event times we have used Efron’s approximation. We have also stratified each dataset according to other available characteristics (e.g., cancer subtype, gender, etc.) to investigate the association of each metagene with patient survival for each subset.

## Results

### Identification of immune gene clusters across five tumor types

To facilitate the de novo discovery of immune-related gene signatures in solid tumors, we assembled microarray datasets of tumor expression profiles for breast, colon, lung, ovarian and prostate cancers from public data repositories. The datasets ranged from 332 to 2,034 tumor profiles and consisted of 22,277 probe sets common to the Affymetrix microarray platforms used. For each dataset, we independently identified all major patterns of co-expressed genes using the EPIG algorithm [16] and an iterative sampling procedure to ensure robustness of gene selections (see Methods: Data filtering using EPIG). Next, the resulting gene patterns (i.e., gene clusters) were systematically analyzed for gene ontology enrichment to identify those significantly enriched for immunity-related terms. The union of all genes comprising immune-enriched clusters (across all 5 datasets) resulted in 1,017 probe sets. The expression patterns of these probe sets were further assessed within each dataset by consensus clustering methodology, i.e., a resampling technique that provides quantitative evidence of cluster stability and enables determination of the number and composition of gene clusters within a dataset [26]. Of note, a variant of this method was used for our initial pattern extraction via EPIG as described in Methods: Data filtering using EPIG.

#### SOM and *k*-means consensus clustering results

**Fig. 1:**
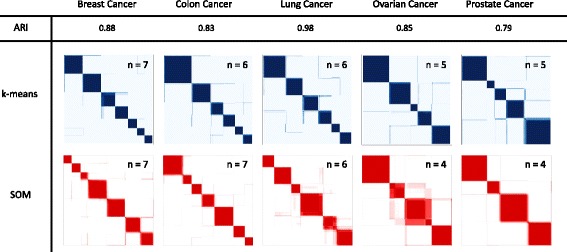
Consensus clustering heatmaps and adjusted Rand index. Consensus matrices are represented as color coded heatmaps. Each entry in the matrix is between 0 and 1, thus we associate a color gradient to the (0,1) range of real number. For *k*-means algorithm 0 = white and 1 = blue, while for SOM 0 = white and 1 = red. A matrix corresponding to perfect consensus is displayed as a color-coded heatmap characterized by blue/red blocks along the diagonal. Numbers inside of each heatmap represent number of clusters selected for each algorithm and dataset. Adjusted Rand index (ARI) is also shown, which measures the agreement between two clustering algorithms with 1 corresponding to perfect agreement. High values for ARI indicate high level of agreement

Within each tumor dataset, the consensus clustering procedure, using both *k*-means and self-organizing map (SOM) clustering algorithms, was performed on the 1,017 probe sets (see methods: Consensus clustering). Analysis of the consensus summary statistics indicated that the optimal number of gene clusters ranged from 5 to 7 by *k*-means clustering, and from 4 to 7 by SOM clustering, depending on cancer type. The adjusted Rand index (ARI), which measures the similarity between two clustering approaches, indicated strong agreement between the two algorithms. The consensus heat maps for the selected gene clusters and adjusted Rand index are displayed in Fig. 1. Additional heatmaps for each dataset and algorithm, and other summary statistics can be found in Additional files 1 and 2.

#### Intersection of clusters and immune gene signatures selection

To identify immune-related gene signatures that are preserved across the five tumor datasets, we compared the gene composition of clusters across the datasets by computing all possible points of cluster intersection. For clarity, by the intersection of two sets *A* and *B*, denoted by *A*∩*B*, we mean all elements of *A* that also belong to *B*. Thus, if *B*_*i*_,*C*_*j*_,*L*_*k*_,*O*_*l*_ and *P*_*m*_ represent specific clusters of probe sets for breast, colon, lung, ovarian and prostate datasets, respectively, then we computed all possible combinations of the following form *B*_*i*_∩*C*_*j*_∩*L*_*k*_∩*O*_*l*_∩*P*_*m*_. In this manner, we had 6,300 intersections for *k*-means and 4,704 intersections for SOM. Next, we narrowed our selection to only the intersections that contained at least ten probe sets, which resulted in 21 intersections for *k*-means and 24 for SOM. Lastly, we combined the results of the two algorithms to generate a meta-consensus, i.e., we chose only the probe sets in common between the 21 *k*-means and 24 SOM intersections. This resulted in 23 final *meta-intersections*, each comprising at least ten probe sets.

**Fig. 2:**
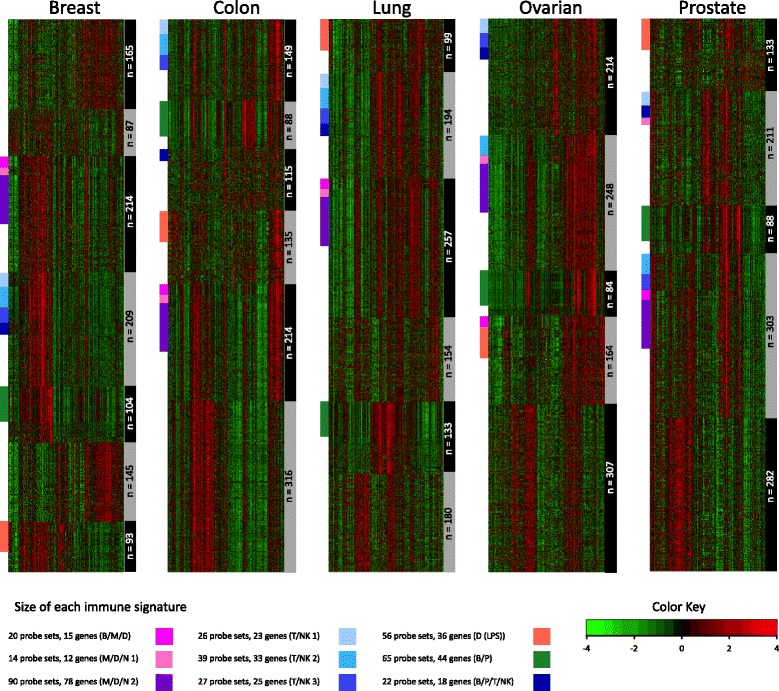
*k*-means clustering and immune gene signatures. Each heatmap represents consensus clustering for *k*-means algorithm. The clusters are represented by gray and black bars on the right-hand side of each heatmap with their respective sizes (number of probe sets) written over gray/black bars. The final nine immune gene signatures are represented by colored bars on the left-hand side of each heatmap

**Fig. 3:**
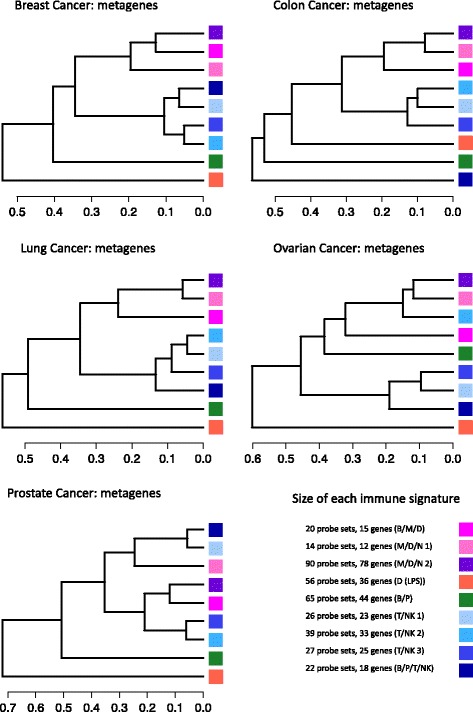
Dendrograms of metagenes. For each dataset, metagenes were hierarchically clustered using Pearson correlation as distance and average linkage. The results were plotted as dendrograms. Each metagene was constructed as described in the Methods section

As a final qualification of immune relevance, gene-annotation enrichment analysis [17, 18] was performed on these 23 meta-intersections, individually (see Methods section and Additional file 3). Nine of the meta-intersections exhibited significant enrichment (FDR <0.05) for terms related to immune cell functions, thereby fulfilling our criteria for *conserved immune gene signatures* in solid tumors. The expression dynamics of the immune gene signatures are shown in Fig. 2. To investigate the correlation structure of the immune gene signatures, we collapsed each signature into a single metagene value (described in Methods) and computed all pairwise correlations within each tumor dataset. As expected, metagenes belonging to the same larger original gene cluster remained highly correlated and primarily grouped together (Fig. 3).

### Immune gene signatures differentiate specific leukocyte populations

**Fig. 4:**
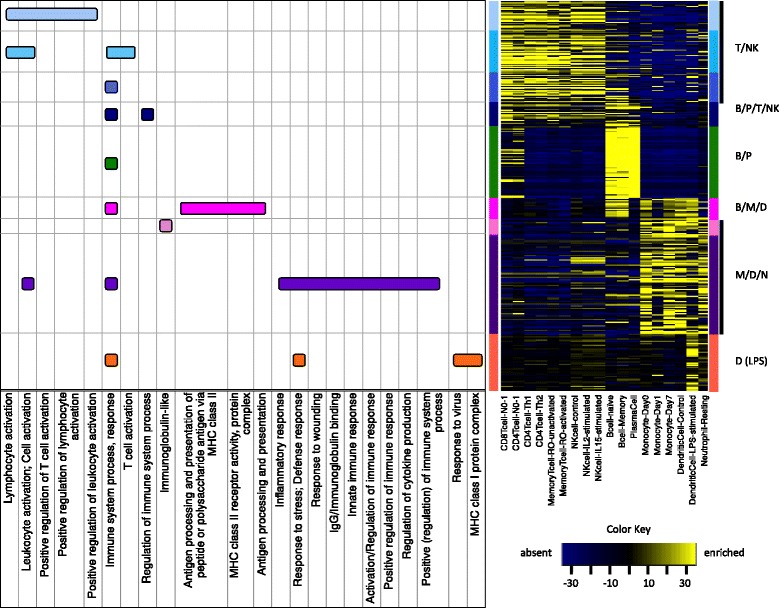
Enrichment scores heatmap and Functional Annotation terms for each immune-signature. Dataset of Abbas et al. [29] was used to compute and visualize enrichment scores as described in Methods section. Major functional annotation terms were determined using DAVID [17, 18]

To investigate the hypothesis that our nine immune gene signatures reflect subpopulations of tumor-infiltrating immune cells, we examined the cellular enrichment of our immune signature genes within a comprehensive collection of leukocyte gene expression profiles (Abbas et al. [29]). Using the Abbas dataset (Table 1), we computed global immune cell type-specific gene enrichment scores [30] (see Methods) then examined the enrichment profiles of our immune gene signatures across the different immune cell types (Fig. 4).

We observed that the immune gene signatures naturally fall into six discrete groups. The first three signatures show strong enrichment in T cells and Natural Killer (NK) cells, and are thus classified here as T/NK. Genes comprising the T/NK signatures include those with conserved roles in T-cell receptor signaling such as TRAC, TRBC1, CD3D, CD3G, TRAT1, CD2, CD7, CD28, LCK and CD247, as well as genes with more specialized roles in activated cytotoxic T lymphocytes (CTLs) including CD8A, PRF1, CCL5, CXCL9, GZMB, GZMA, GZMH, GZMK, CTSW, IL2RB and CRTAM. One signature, termed B/P/T/NK exhibited a broader lymphocytic enrichment characteristic of B cells, plasma B cells, T cells and NK cells. It includes B cell signaling genes such as CD19, CD79A and CD180, and genes involved in lymphocyte differentiating and trafficking including IKZF1, CXCR3, IL16 and ITGB7. One signature, termed B/P, is strongly enriched in B cells, and plasma B cells in particular, and is composed primarily of immunoglobulin-encoding genes such as IGKC, IGHD, IGLC1, IGLJ3, IGHA1, IGHM, IGJ and IGK. One signature, termed B/M/D, is enriched in B cells, monocytes and dendritic cells, and is predominated by genes that belong to the major histocompatibility complex (MHC) class II family (HLA-DRA, HLA-DRB1, HLA-DPA1, HLA-DPB1, HLA-DQB1, CD74) consistent with roles in professional antigen presentation. Two gene signatures, termed M/D/N, are enriched in monocytes, dendritic cells and neutrophils. These signatures comprise genes involved in the activation and recruitment of effector lymphocytes (CD84, CD86, CCR1), regulation of immune responses (LILRB2, LILRB4, CD300A), macrophage differentiation and function (CSF1R, CCL2, CD14, CD163, CYBB, CLEC4A, CLEC7A) and myeloid IgG receptor signaling (FCER1G, FCGR1A, FCGR1B, FCGR2A, FCGR2B, FCGR3A, FCGR3B). Finally, one gene signature, termed D (LPS), showed greatest enrichment in LPS-stimulated dendritic cells and is composed of major histocompatibility complex (MHC) class I family genes (HLA-B, HLA-C, HLA-G, HLA-J) and a large number of genes with direct roles in interferon signaling (IRF7, IRF9, STAT1, ISG15, OAS1, OAS2, OAS3, IFI35, IFI44, IFI6, IFIH1, IFIT3, IFIT5, HERC5, HERC6, DDX58, DDX60). Gene symbols associated with each signature are listed in Table 2 (genes that had no symbol or had more than three symbols representing the same probe are listed with Affy Probe ID).

**Table 2:** Immune gene signatures and gene symbols

Gene signature	Gene symbol
T/NK 1	BIN2; CD3G; CD7; CD72; CTSW; CXCR6; FYB; GZMH; IKZF1; IL21R; KLRC4-KLRK1/KLRK1; KLRD1; LCK; MAP4K1; PSTPIP1; PTPN7; SIRPG; SP140; STAT4; TRAF1; UBASH3A; YME1L1; ZAP70
T/NK 2	ARHGAP25; CCL5; CCR5; CD2; CD3D; CD48; CD8A; CORO1A; CST7; CXCL9; FYB; GIMAP1-GIMAP5/GIMAP5; GZMA; GZMB; GZMK; IL2RB; ITGAL; LCK; LTB; NCF1/NCF1B/NCF1C; NCF1C; NKG7; PIK3CD; PRF1; RASGRP1; SASH3; SELL; TNFAIP3; TRAC; TRAC/TRAJ17/TRAV20; TRAF3IP3; TRBC1; YME1L1
T/NK 3	ACAP1; ARHGAP25; CCL19; CCR7; CD247; CD28; CD96; CRTAM; FAIM3; GPR171; GPR18; HLA-DOB; IL16; KLRB1; LAT; LRMP; MS4A1; PLCG2; PPP1R16B; PVRIG; RUNX3; SH2D1A; TRAT1; XCL1; XCL1/XCL2
B/P/T/NK	CD180; CD19; CD79B; CD8B/LOC100996919; CXCR3; DENND1C; FAIM3; IKZF1; IL16; ITGB7; LAT; LY9; PAX5; PLA2G2D; PRKCQ; SH2D1A; SIT1; TCL1A
B/P	217179_x_at; 211633_x_at; 217480_x_at; 211868_x_at; 211637_x_at; 211639_x_at; 217281_x_at; 211650_x_at; 211635_x_at; 211641_x_at; 214916_x_at; 216557_x_at; 217360_x_at; 216510_x_at; 211430_s_at; 216401_x_at; 214768_x_at; 216984_x_at; IGHD; IGHM; IGJ; IGK; IGHG1/IGHM; IGHV3-47/IGHV3-47; IGK/IGKC; IGKC; IGKV1-17/IGKV1-17; IGHA1/IGHG1/IGHM; CYAT1/IGLC1/IGLV1-44; GUSBP11; IGH/IGHA1/IGHA2; IGKV4-1/IGKV4-1; IGLC1; IGLJ3; IGLL3P; IGLL5; IGLV1-44; IGLV2-14/IGLV2-14; IGLV3-10/IGLV3-10; IGLV3-19/IGLV3-19; MZB1; PIM2; TNFRSF17; AC016745.2/OTTHUMG00000153338;
B/M/D	215193_x_at; 209312_x_at; 204670_x_at; CD74; HLA-DPA1; HLA-DPB1; HLA-DQB1; HLA-DRA; SLC15A3; SLC1A3; THEMIS2; TMEM140; HLA-DRB1/LOC100507709/LOC100507714; HLA-DQB1/LOC101060835; HLA-DRB6/LOC100996809
M/D/N 1	APOC1; CCL18/LOC101060271; CCR1; CD300A; CD84; CD86; FCGR2C; LILRB2; LILRB4; LSP1; PILRA; SLAMF8
M/D/N 2	219574_at; AIF1; ALOX5AP; APOE; BCL2A1; C1QA; C1QB; C3AR1; C5AR1; CCL2; CCL4; CCR1; CD14; CD163; CD300A; CD53; CD86; CLEC2B; CLEC4A; CLEC7A; CSF1R; CYBB; DOCK10; DOCK2; EVI2A; EVI2B; FCER1G; FCGR1A/FCGR1B/FCGR1C; FCGR1B; FCGR2A; FCGR2B; FCGR3A/FCGR3B; FCGR3B; FGL2; GPNMB; GPR65; HCK; HCLS1; IFI30/PIK3R2; IGSF6; ITGA4; ITGB2; LAIR1; LAPTM5; LCP2; LILRB2; LST1; LY86; LY96; MNDA; MRC1; MS4A4A; MS4A6A; MSR1; MYO1F; NCF2; NCKAP1L; NPL; PILRA; PLEK; PTPRC; RGS1; RNASE6; SAMSN1; SELPLG; SLA; SLAMF8; SLC7A7; SLCO2B1; SRGN; TFEC; TLR1; TLR2; TLR7; TM6SF1; TRPV2; TYROBP; VSIG4
D (LPS)	B2M; CXCL10; CXCL11; DDX58; DDX60; EIF2AK2; HERC5; HERC6; HLA-B; HLA-C; HLA-G; HLA-J; IFI35; IFI44; IFI44L; IFI6; IFIH1; IFIT1; IFIT3; IFIT5; IRF7; IRF9; ISG15; LAP3; OAS1; OAS2; OAS3; PLSCR1; PSMB8; RSAD2; STAT1; TAP1; TAPBP; UBE2L6; USP18; WARS

Genes without symbol or with more than three symbols per probe are listed with Affy Probe ID

### Most immune gene signatures exhibit minimal and uncorrelated expression in cancer cell lines derived from solid tumors

**Fig. 5:**
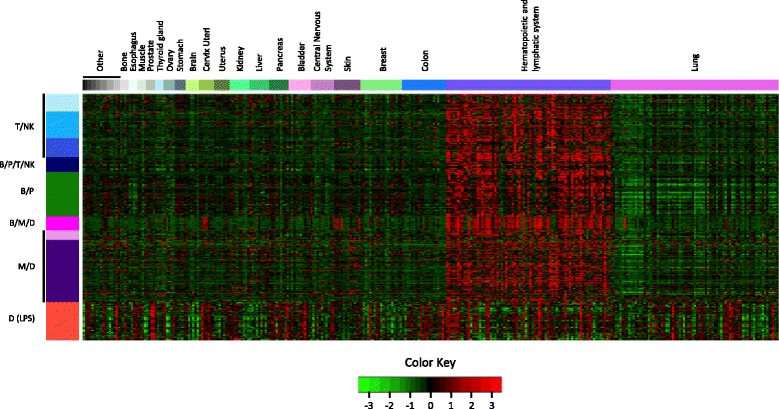
GSK cancer cell lines and immune gene signatures. Cell lines were arranged by cancer type and are represented by the colored bar at the top of the heatmap. There are 318 cancer cell lines representing 28 different cancer types. Cancer types labeled *Other* are (in order from left to right): Eye, Synovial Membrane, Pharynx, Rectum, Sarcoma, Connective Tissue, Placenta, Vulva. Samples and immune gene signatures were not clustered

To further investigate the hypothesis that our nine immune gene signatures reflect subpopulations of tumor-infiltrating immune cells, we examined the expression patterns of the immune signature genes in a microarray dataset provided by GlaxoSmithKline (GSK) (see Methods for details) which comprises of >300 cancer cell lines derived from solid tumors (*n*=243) and hematopoietic and lymphatic cancers (*n*=75) representing 28 different cancer types. Shown in Fig. 5 is a heat map that displays the relative gene expression levels of our nine immune gene signatures. Consistent with immune-restricted expression, the majority of the signature genes displayed a significantly heightened expression in cancer cell lines of hematopoietic and lymphatic (immune cell) origin (i.e., lymphomas, leukemias and myelomas). By contrast, expression of the immune signature genes in cell lines derived from solid tumors tended to exhibit markedly reduced and uncorrelated expression patterns, consistent with the notion that cancer cell lines cultured from solid tumors are immune deficient. However, two exceptions were observed. The B/M/D signature, comprising largely of genes encoding MHC class II antigen presenting molecules, showed enhanced expression in several solid tumor types, most notably cancers of the skin (melanomas) and cervix. Indeed, the overexpression of these genes is well documented in multiple epithelial cancers, most notably melanoma [40, 41] and cervical cancer [42, 43], though its pathological contributions are not known. Contrary to the other immune signatures, the D (LPS) signature, comprised mainly of interferon-regulated genes, displayed marked up-regulation in a portion of all cancer cell types. Not surprisingly, the majority of these genes have been previously defined as components of a conserved interferon activation signature, observed not only in various cancers [44–47], but also autoimmune diseases [48, 49]. Thus, we conclude that, with the exception the latter two signatures, the tumor immune gene signatures identified here likely derive, in large part, from the infiltrating immune component of the tumor microenvironment.

### The immune gene signatures are robust prognostic markers

**Fig. 6:**
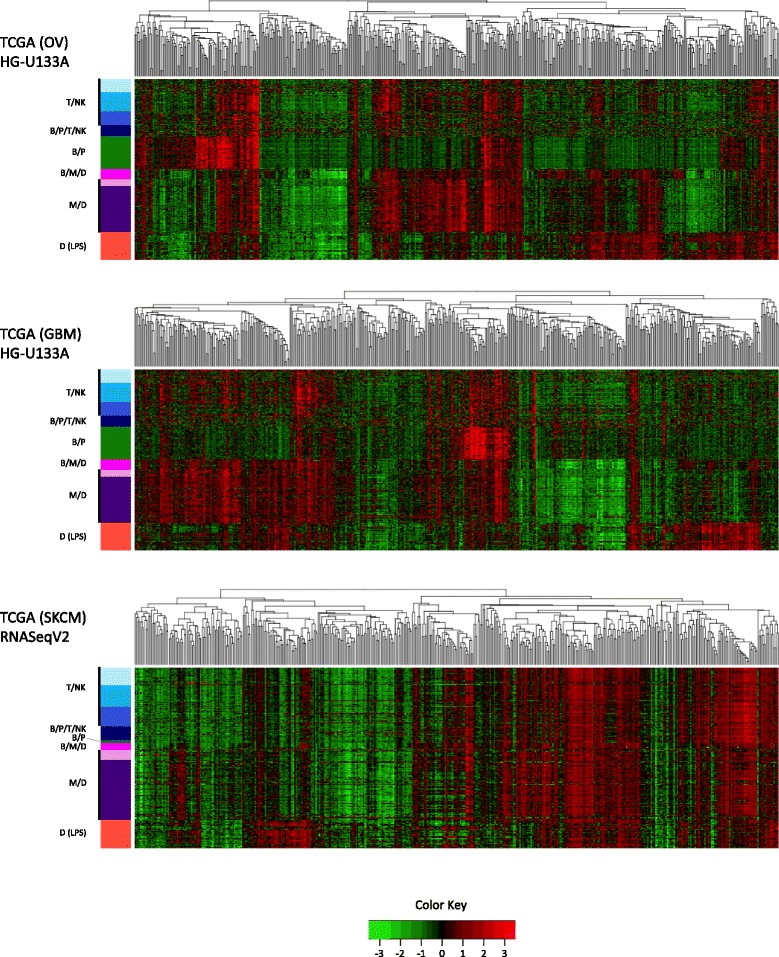
TCGA datasets and immune gene signatures. Samples for each dataset (OV, GBM and SKCM) were hierarchically clustered using Pearson correlation as distance and average linkage. Gene signatures were not clustered

Next, we examined the extent to which the nine immune signatures (i.e., metagenes) associate significantly with patient prognosis. Since the immune signatures were discovered independently of the clinical outcome data, our statistical analysis utilized three subsets from our original datasets (breast, colon and lung) and three independent TCGA (http://cancergenome.nih.gov/) datasets: Glioblastoma multiforme (GBM), Ovarian serous cystadenocarcinoma (OV) and Skin Cutaneous Melanoma (SKCM) (see Methods section on Datasets for statistical analyses details). Prior to survival analysis, we investigated whether the discovered signatures display similar patterns of gene correlation structure when applied to TCGA OV, GBM, and SKCM data. As shown in Fig. 6, the genes comprising the nine immune signatures do in fact retain a preserved intra-signature co-expression structure in all three TCGA datasets.

To assess associations with overall and/or recurrence- or progression-free survival, we performed univariate Cox proportional hazards regression using the immune metagenes as continuous explanatory variables. For each tumor dataset, we performed multiple survival analyses based on the differential stratification of patients according to a variety of potentially relevant clinical and biological tumor characteristics; the latter of which included a tumor proliferation metagene (P metagene) that we previously demonstrated in breast cancer to markedly influence the prognostic strength of several immune metagenes upon stratifying patients to different P metagene tertiles [9]. Numerous significant results were observed and are presented in Table 3 (for the entire summary that includes hazard ratios and 95% confidence intervals see Additional file 4). As the table demonstrates, all nine immune metagenes achieved statistically significant associations with DMFS (distant metastasis-free survival) and/or OAS (overall survival), with greatest positive significance (i.e., high immune metagenes associated with good outcomes) observed in the Breast and SKCM cancer types. By contrast, however, a number of metagenes exhibited inverse survival associations under various circumstances. This poor-outcome association was most apparent for metagenes enriched in myeloid cells and occurred most notably in the contexts of GBM and colon cancer. Together, these findings are consistent with the perception that tumor infiltrating immune cells possess the functional capacity to promote both anti- and pro-tumorigenic effects, where the directionality and extent of effect is governed, in part, by cellular and molecular constituents of the tumor microenvironment that vary within and across tumor types.

**Table 3:** Significant *p*-values from univariate survival analysis of immune metagenes

		Immune metagenes								
Cancer type	Clinical characteristics	T/NK 1	T/NK 2	T/NK 3	B/P/T/NK	B/P	B/M/D	M/D/N 1	M/D/N 2	D (LPS)
Breast (DMFS)	All (*n*=1954)	**	**	**	**	***	***			
	P (high) (*n*=651)	***	***	***	***	***	***	***	***	**
	HER2-E (*n*=281)	***	***	***	***	***	**	**	*	**
	Basal (*n*=334)	***	***	***	***	***	**	**		
	LN- (*n*=1498)	**	**	**	**	***	***			
	ER- (*n*=401)	***	***	***	***	***	**			
	ER+ (*n*=1343)			*		**	***			* *↓*
	Luminal B (*n*=399)		*	*		*	***		*	
	Luminal A (*n*=565)									* *↓*
	Normal (*n*=257)					*				
	40<Age≤50(*n*=404)			*	*	**	**			
	Age>50(*n*=986)			*		*	*			
Colon (OAS)	All (*n*=232)				**					
	Grade - MD (*n*=166)	*			*					
	Age <60(*n*=79)	*			*					
	P (low) (*n*=78)				*					
	Gender - M (*n*=122)	*	*	*	**					
	Gender - F (*n*=110)									* *↓*
	Stage II (*n*=72)		* *↓*			* *↓*	* *↓*	** *↓*	** *↓*	** *↓*
Lung (OAS)	All (*n*=757)		*	**	*	**				
	Stage I (*n*=490)		**	**	*	**				
	Sub-stage IB (*n*=313)	*	**	**	*	**			*	
	Adj. Chemo - No (*n*=503)		*	**	*	*				
	Histology - S (*n*=183)		*	*		**				
	Histology - A (*n*=574)			*						
	60≤Age<70(*n*=273)		*	**			*			
	P (med) (*n*=253)			*		*				
	Gender - F (*n*=333)			*						
	Gender - M (*n*=424)					*				
TCGA OV (OAS)	All (*n*=566)	*	*	*	*					
	P (med) (*n*=189)	**	**	**	*	*	*	*		
	P (low) (*n*=188)						* *↓*		* *↓*	
	Grade 3 (*n*=477)	**	*	**	*					
	Stage III (*n*=436)	*		*	*					
	60≤Age<70(*n*=136)				*					
TCGA GBM (OAS)	All (*n*=524)							* *↓*	* *↓*	* *↓*
	Age <60(*n*=273)							* *↓*	* *↓*	** *↓*
	Gender - F (*n*=205)	* *↓*	* *↓*			* *↓*	* *↓*	** *↓*	** *↓*	
	P (high) (*n*=174)		* *↓*				** *↓*	** *↓*	** *↓*	* *↓*
TCGA SKCM (OAS)	All (*n*=456)	***	***	***	***	***	***	***	***	***
	Stage ≤ II (*n*=229)	***	***	***	***	***	***	***	***	***
	Stage > II (*n*=191)	***	***	**	**	**	***	**	***	***
	Gender - F (*n*=175)	***	***	***	**	***	***	***	***	***
	Gender - M (*n*=281)	**	**	*	**	*	**	**	**	**
	P (low) (*n*=152)	**	**	**	*	**	**	**	***	***
	P (med) (*n*=152)	*	*				**	*	*	**
	P (high) (*n*=152)	**	**	*	**	**	**	**	**	**
	Age<50(*n*=136)	**	**	*	*	*	*			**
	50≤Age<70(*n*=190)	**	**	*	*	**	***	***	***	**
	Age≥70(*n*=130)	**	**	*	**	*	**	*	*	**
	Breslow dv ≤1.5(*n*=105)	***	**	**	*	*	***	**	***	**
	Breslow dv >1.5(*n*=246)	*				*	*		*	*
	Primary Tumor (*n*=102)									*
	Regional Tumor (*n*=288)	***	***	***	***	***	***	***	***	***

*p*-value codes: *p*≤0.001 (***); 0.001<*p*≤0.01 (**); 0.01<*p*≤0.05 (*); Poor outcome (*↓*)

P (low, med, high) = low, intermediate and high proliferation tertiles. Breslow dv (depth value)

Grade - MD = Moderately Differentiated. Histology - S/A = Squamous Cell Carcinoma/Adenocarcinoma

## Discussion

A number of expression profiling studies have demonstrated the existence of a relationship between intratumoral immune gene signatures and favorable prognosis or response to therapy, either chemotherapy or immunotherapy [50–52]. Although overlapping biological properties characterizing the favorable cancer immune phenotype have been described [50], the gene makeup of these signatures lacks consensus, the cellular specificity of the gene expression signals are unknown and a systematic analysis of their prognostic value within multiple tumor types is lacking. Only very recently, an integrative meta-analysis has corroborated the prognostic role of immune gene signatures across cancer [53]. In this study, we instituted a de novo discovery approach to rigorously identify co-expressed genes enriched for immune cell function and conserved in correlation structure across anatomically diverse malignancies. We hypothesized that the existence of such gene signatures could be explained by gene expression patterns specific to infiltrating immune cells with negligible transcriptional contribution from cancer cells or other stromal compartments that would otherwise disrupt the conserved internal correlation among the genes comprising the signatures. As quantifiable surrogates of tumor infiltrating immune cells, we further posited that the immune gene signatures (quantified as metagenes) would significantly associate with measures of disease aggressiveness such as tumor recurrence and patient survival in a manner typifying the functional attributes of distinct immune cell lineages in anti- or pro-tumor immunity.

Using unsupervised and consensus clustering methods followed by assessment for enrichment of immunological processes, we identified 9 distinct gene signatures conserved across breast, colon, lung, ovarian and prostate cancers that appear to reflect different functional aspects of immune cell biology. Enrichment analysis of their patterns of expression in blood-purified immune cell lineages (Fig. 4) revealed large distinctions between lymphoid and myeloid tissues, but with limited resolution among more specific immune cell types, with the exception of a highly specific B cell/Plasma cell (B/P) gene module shared by naïve, memory and plasma B cells. Notably, the genes of this signature have been previously recognized in a number of independent studies, overlapping substantially with prognostic and therapy-predictive B-cell signatures in breast cancer [9, 54–56], an IgG metagene in breast cancer [57] and a gene signature of B-cell TILs in breast and ovarian tumor subtypes associated with prognostic low-diversity B-cell receptor (BCR) gene segments [58]. A signature termed D (LPS) that showed strongest enrichment in LPS-stimulated dendritic cells, provided little distinction between lymphoid and myeloid tissues, generally, and in contrast to the other gene signatures, showed relatively similar expression levels across the entirety of immune cell types. These results suggest that while the gene signatures can largely distinguish immune lineages from the common progenitors (lymphoid and myeloid), and also B cells with marked specificity, the more differentiated cells that stem from a common developmental precursor (e.g., CD8+ T cells, CD4+ T cells, CD56+ NK cells) are largely not discernible by the immune gene signatures. There are several possible explanations for this lack of cell type-specific resolution. First, a comparative analysis of the global gene expression profiles of the purified immune cells revealed a moderate to high degree of transcriptional similarity among differentiated cells related by lineage, with only a few genes, in some instances, exhibiting robust cellular specificity [29]. That these rare, cell type-specific genes were not major components of our gene signatures could owe to their admixed expression in tumors, where both immune and malignant cells (and/or other stromal cell populations) may co-express the genes thereby abrogating their cellular specificity that otherwise exists among peripheral blood-purified immune cells. Second, it is currently unknown to what extent immune cells from peripheral blood share transcriptional programming with immune cells residing in the tumor microenvironment. Secreted factors unique to this environment could induce systemic transcriptional alterations in tumor infiltrating immune cells that, while contributing to cellular specificity underlying the tumor-derived immune gene signatures, may not accurately reflect cellular identity in peripheral blood counterparts. Third, the derivation of the immune gene signatures required a sufficient number of genes per signature to achieve statistical significance for enrichment of immune-related processes. Thus, a relatively small (and immune enrichment-insignificant) number of correlated and conserved cell-specific genes could have been precluded by our statistical selection criteria.

Consistent with their immunological origins, a positive correlation between immune signatures (i.e., metagenes) and good prognosis was observed in all but GBM tumors, in which the expression of the immune metagenes associated with B cell, monocyte and dendritic cell infiltration was inversely correlated with outcome. Studies assessing the prognostic role of immune signatures in GBM have reported contrasting results [59–61]. However, differential expression of immune genes according to GBM molecular subtypes has been described [62]. As intrinsic molecular subtypes of GBM in turn associate with different clinical outcomes, further analyses should clarify the role and functional orientation of immune infiltrates within specific GBM molecular subtypes. While our study did not uncover immune metagenes that consistently showed negative correlations with patient outcomes, the myeloid-like signatures exhibited the greatest variation in direction of prognosis (good versus poor), which appears to depend on cancer type and specific diagnostic contexts. This finding may exemplify the myriad and opposing pathological roles played by myeloid cells in cancer.

The transcriptional profiling of whole tumor specimens cannot clarify the source of immune-signature signals. To further address the question of immune cellular specificity of gene expression, we examined the expression profiles of the immune gene signatures across several hundred cancer cell lines (Fig. 5). Under baseline conditions, solid tumor cells generally displayed negligible expression of the immune signature genes. By contrast, the genes tended to exhibit highest expression in hematopoietic and lymphatic cancers, consistent with the hypothesis that immune signals detected from whole tumor samples are mostly driven by the presence of immune cell infiltrates. However, two notable exceptions were observed. The D (LPS) signature exhibited substantial expression heterogeneity in solid and liquid cancer cell lines. This signature is enriched for interferon-regulated genes such as transcripts coding for classical IFN-induced chemokines (e.g., CXCL9 and CXCL11), and other IFN-regulated transcripts (e.g., STAT1, IRF7, IRF9, STAT1, ISG15, OAS1, OAS2, OAS3, IFI35, IFI44, IFI6, IFIH1, IFIT3, IFIT5, HERC5, HERC6, DDX58, and DDX60). It has been observed that the degree of T cell infiltration in ovarian cancer correlated with the expression of the interferon regulatory factor IRF1, the major transcriptional activator of genes induced by alpha, beta and gamma interferons. Positive staining of IRF1 was predominantly observed in ovarian cancer cells (cell lines and tumors) with lesser but detectable expression observed in tumor infiltrating lymphocytes [63]. Interferon signaling has well established roles in both immunological and non-immunological tissues (including epithelium) where it elicits diverse cellular responses. Interferon signaling is activated in many tissue types in response to viral and bacterial infection. In cancer, interferon signaling is the main mediator of immune-surveillance mechanisms, and its activation is critical for the development of immune-mediated rejection. However, it is also responsible for the activation of counter-regulatory/pro-tumorigenic immune mechanisms [51, 64]. Similarly, but to a lesser extent, the B/M/D signature genes exhibited tandem elevation in solid cancer cell lines derived from melanomas, cervix and lung cancers consistent with up-regulation of MHC class II antigen presenting molecules in malignant melanoma [40, 41] and cervical cancer [42, 43]. Together, these observations suggest that the D (LPS) and B/M/D signatures integrate transcriptional signals from both immune and malignant cell compartments, while the other immune signatures are relatively immune-specific in their expression.

That each of the immune signatures exhibited significant prognostic value in multiple cancer types lends credence to the concept of an ‘immune grading index’ for assessing patient prognosis based on combinations of the immune gene signatures. The application of such an index would require further investigation involving multivariate modeling to determine the independent and additive value of the signatures in combination, as well as in the context of different cancer types, where our findings suggest differential tuning would be required for maximal prognostic results. To the extent to which these immune signatures reflect the functional orientation of infiltrating immune cell populations, it is logical that their prognostic information could be predictive of therapeutic outcomes as well, particularly for treatments where efficacy depends on immune system response, such as current and emerging immunotherapy approaches. The clinical merit of such applications will be the focus of future studies.

## Conclusions

Our results are the first to identify a diversity of immune gene signatures that are robustly conserved across solid tumor types. At the core of our immune signatures are genes that reflect specific immunological functions and broadly distinguish immune cell populations. We show that the immune signatures exhibit robust prognostic associations that vary between lymphocytic and myeloid signatures and according to cancer type. Looking ahead, our findings suggest that the immune signatures described here could form the basis of a tumor-agnostic immunological grading index for assessing patient prognosis or predicting response to immune-modulating therapies.

## Additional files

Additional file 1 Consensus clustering summary of *k*-means algorithm. This Excel file contains summary of the *k*-means consensus clustering results as produced by *clusterCons* R package [12, 28]. Each cancer dataset (breast, colon, lung ovarian and prostate) has two tabs. One tab displays images of the summary statistics. The other tab lists the number of clusters selected for our analysis and contains Affy ID, gene symbol and title together with membership robustness. (XLSX 2170 kb)

Additional file 2 Consensus clustering summary of SOM algorithm. This Excel file contains summary of the SOM consensus clustering results as produced by *ConsensusClustering* GenePattern module [26, 27]. Each cancer dataset (breast, colon, lung ovarian and prostate) has two tabs. One tab displays images of the summary statistics. The other tab lists the number of clusters selected for our analysis and contains Affy ID, gene symbol and title. (XLSX 5007 kb)

Additional file 3 Combined meta-intersections between two algorithms SOM and *k*-means. This Excel file contains final 23 meta-intersections as described in Results section. Each intersection is in separate tab, which also contains gene-annotation enrichment analysis results. (XLSX 721 kb)

Additional file 4 Univariate survival analysis. This Excel file contains all results of univariate survival analysis. (XLSX 159 kb)

## Abbreviations

ARI: Adjusted Rand index
BCR: B-cell receptor
CTL: Cytotoxic T lymphocytes
DC: Dendritic cells (DC)
DMFS: Distant metastasis-free survival
DSS: Disease specific survival
DFS: Disease free survival
GBM: Glioblastoma multiforme
IHC: Immunohistochemical
MDSC: Myeloid derived suppressor cells
NK: Natural killer cells
OAS: Overall survival
OV: Ovarian serous cystadenocarcinoma
PFS: Progression-free survival
RFS: Relapse-free survival
RMA: Robust Multi-array Average
SOM: Self-organizing map
SKCM: Skin cutaneous melanoma. T_H_: T-helper cells
TAM: Tumor associated macrophages
TIL: Tumor infiltrating lymphocytes
